# Supplementary material for: A cytomegalovirus inflammasome inhibitor reduces proinflammatory cytokine release and pyroptosis
Source: Nat Commun. 2024 Jan 26;15:786. doi: 10.1038/s41467-024-45151-z (PMC10817922; doi:10.1038/s41467-024-45151-z)
Supplement: Supplementary file 1 — Supplementary Information [file 41467_2024_45151_MOESM1_ESM.pdf]

SUPPLEMENTARY FIGURES

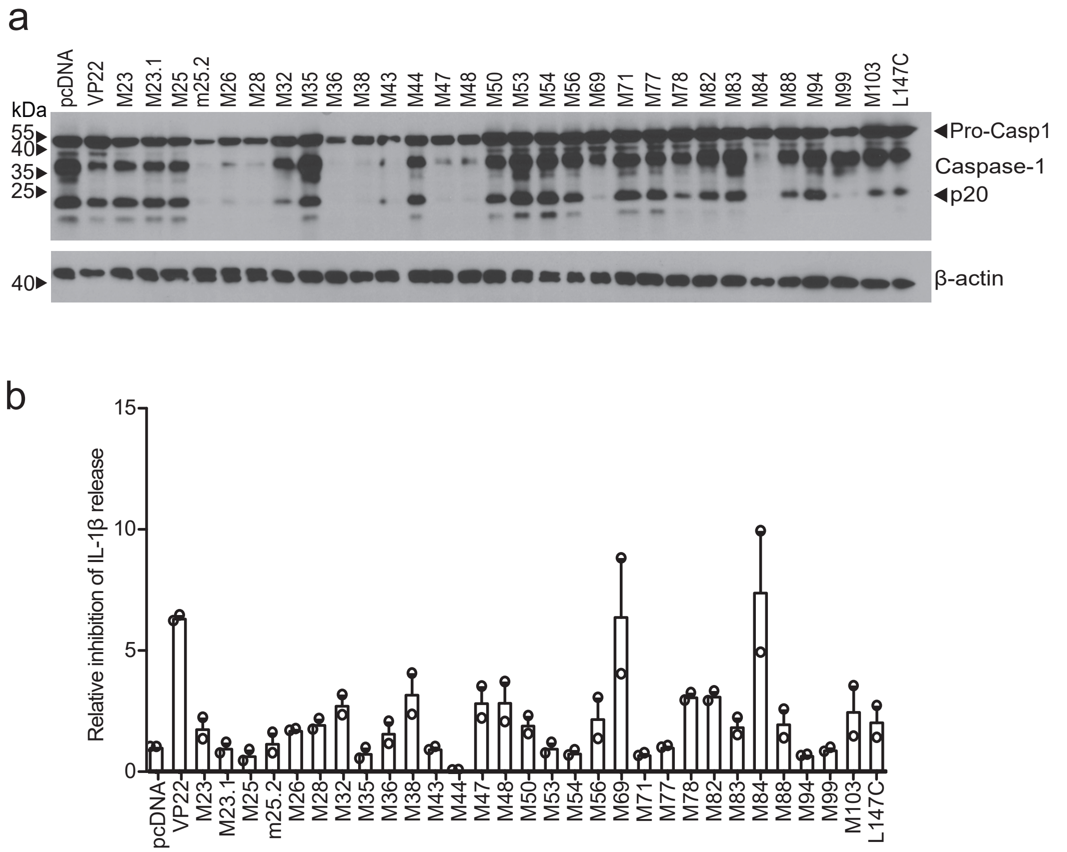

Supplementary Figure 1. Identification of MCMV AIM2 inflammasome inhibitors.

(a and b) HEK 293A cells were co-transfected with plasmids encoding murine AIM2, ASC, pro-Caspase-1, pro-IL-1β, and individual MCMV proteins. 24 h post transfection, cell lysates were harvested to detect Caspase-1 activation by immunoblot analysis. Data are representative of three biologically independent experiments (a). Supernatants were collected to determine IL-1β release by ELISA (b). The inhibitory effect was calculated by dividing the IL-1β level of vector-transfected cells by those from cells expressing MCMV ORFs. Mean ± SEM of two independent experiments are shown.

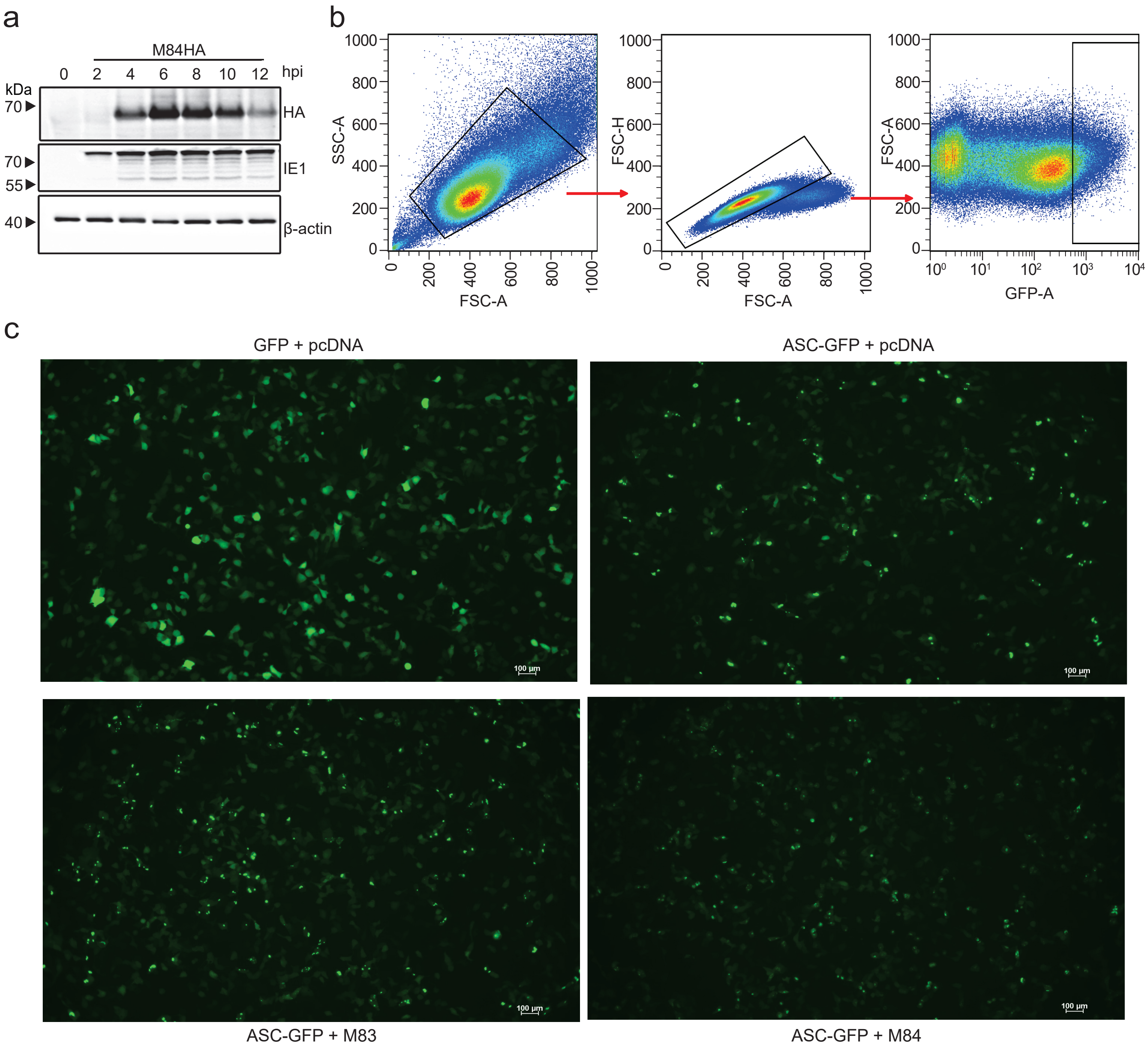

Supplementary Figure 2. The inhibition of ASC speck formation by M84.

(a) Kinetics of M84 expression in iBMDM infected with MCMV-M84HA (MOI=5), analyzed by immunoblot. Data are representative of three biologically independent experiments.(b) Gating strategy for the analysis of speck formation. Transfected HEK 293A cells were gated for live single cells and subsequently for GFP expression. Only the high-level GFP-positive cells were analyzed for ASC speck formation. (c) HEK 293A cells were co-transfected with plasmids encoding AIM2, ASC-GFP or GFP (control), and M83 or M84 or empty vector (pcDNA). Representative images taken by fluorescence microscopy are shown (scale bar, 100 µm).

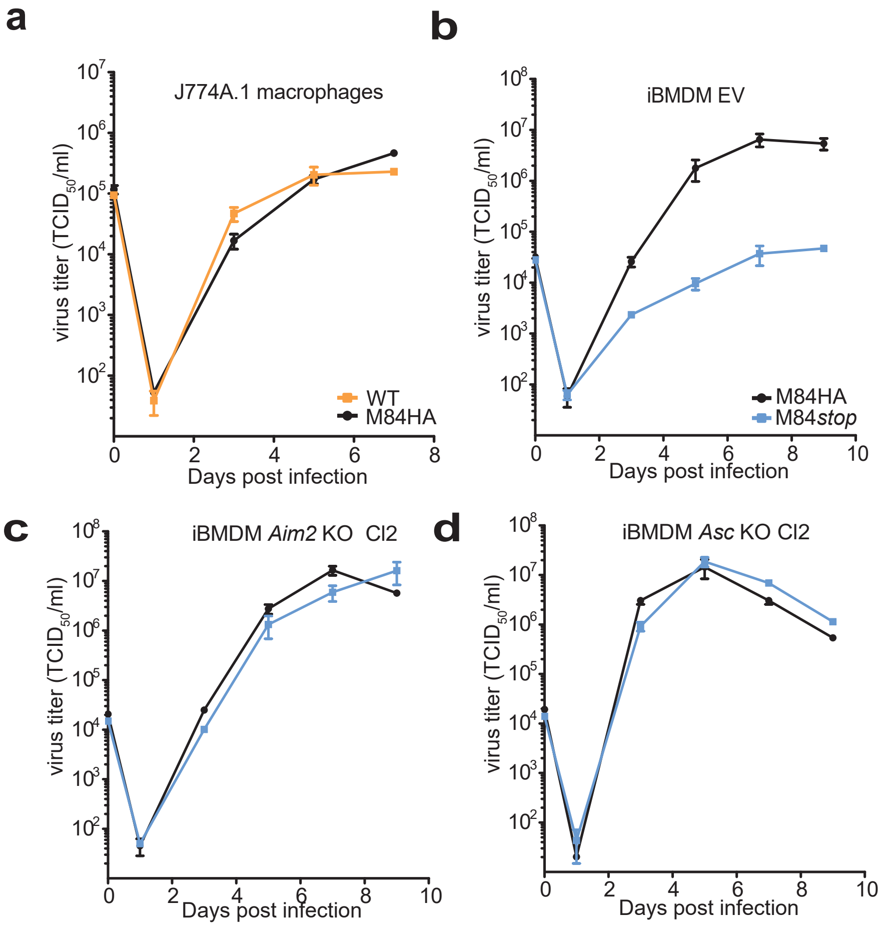

Supplementary Figure 3. Replication of M84*stop* MCMV in macrophages.

(a) Multistep replication kinetic of WT or M84HA MCMV in J774A.1 macrophages infected at a MOI of 0.5. (b) Multistep replication kinetics of MCMV M84HA and MCMV M84*stop* in empty vector (EV)-transduced iBMDMs, (c) *Aim2* KO iBMDMs (clone 2), and (d) *Asc* KO iBMDMs (clone 2) infected at MOI=0.025. Viral titers are shown as mean ±SEM of three biological replicates.

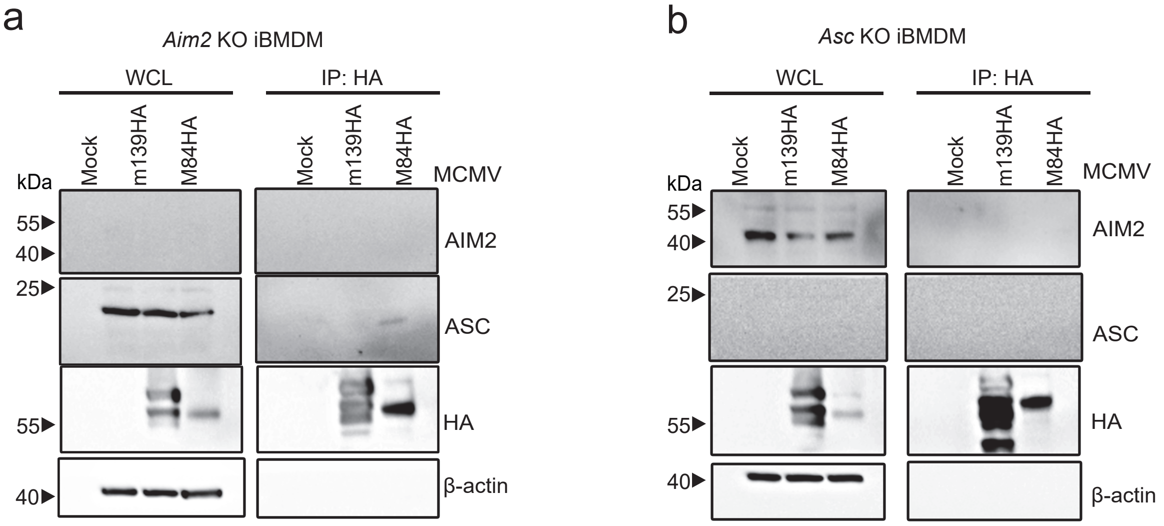

Supplementary Figure 4. The interaction of M84 with AIM2 and ASC is interdependent.

(a) *Aim2* KO and (b) *Asc* KO iBMDMs were infected with MCMV M84HA or m139HA (MOI=5). At 10 h post-infection, cell lysates were collected for co-IP using an anti-HA affinity matrix. Co-precipitating proteins were analyzed by immunoblot with AIM2 and ASC-specific antibodies. Data are representative of three biologically independent experiments.

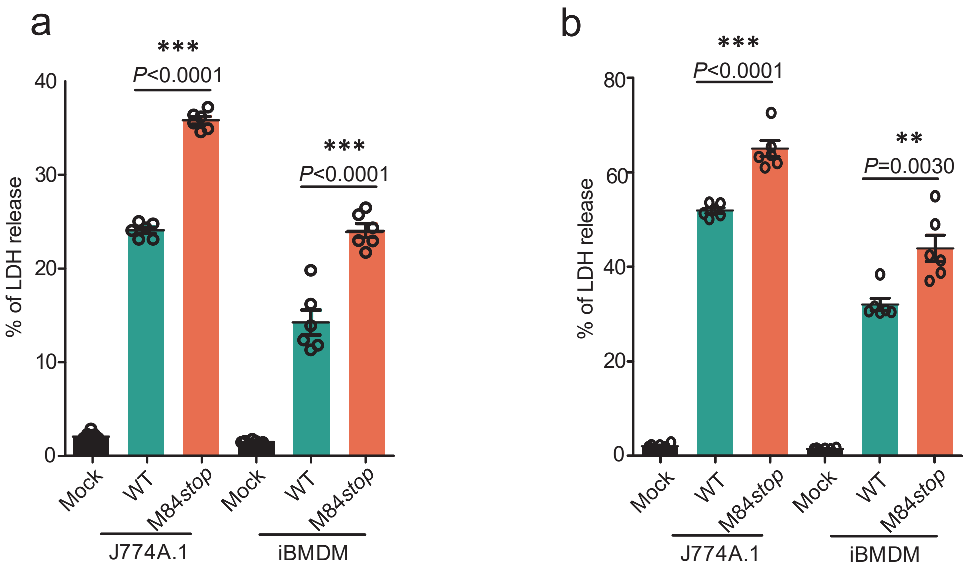

Supplementary Figure 5. LDH release from MCMV-infected macrophages.

J774A.1 macrophages and iBMDM were infected with WT MCMV (green) or M84*stop* (orange) (MOI=3). The release of LDH into the supernatant was analyzed at 7 (a) and 10 hpi (b) with a CytoTox 96 Non-Radioactive Cytotoxicity Assay kit. Mean ± SEM of 6 biological replicates are shown. Significance was determined by using the two-tailed Student's *t*-test. ** $P<0.01$, *** $P<0.001$.

Antibody	Clone	Catalog number	Supplier	WB dilution	IF dilution
Primary:					
HA	3F10	11867423001	Roche	1:1000	1:300
Flag	M2	F3165	Sigma-Aldrich	1:2500	1:300
MCMV IE1	CROMA 101	HR-MCMV-08	Center for Proteomics, University of Rijeka	1:1000	
β-Actin	AC-15	A1978	Sigma-Aldrich	1:10000	
AIM2		63660	Cell Signaling	1:1000	
ASC	D2W8U	67824	Cell Signaling	1:1000	
Caspase-1	Casper-1	AG-20B-0042-C100	Adipogen	1:1000	
GSDMD	EPR19828	ab209845	Abcam	1:1000	
GFP	7.1 and 13.1	11814460001	Roche	1:1000	
Secondary:					
Anti-Rat Alexa Fluor 488		A-21208	Invitrogen		1:1000
Anti-Mouse HRP		P044701-2	Dako	1:5000	
Anti-Rabbit HRP		P039901-2	Dako	1:5000	
Anti-Rabbit IgG heavy chain		ab99702	Abcam	1:4000	
Anti-Rat HRP		112-035-062	Jackson ImmunoResearch	1:5000	

Supplementary Table 1. List of antibodies used in this study
