# Supplementary material for: A cytomegalovirus inflammasome inhibitor reduces proinflammatory cytokine release and pyroptosis
Source: Nat Commun. 2024 Jan 26;15:786. doi: 10.1038/s41467-024-45151-z (PMC10817922; doi:10.1038/s41467-024-45151-z)
Supplement: Supplementary file 3 — Reporting Summary [file 41467_2024_45151_MOESM3_ESM.pdf]

nature portfolio

Corresponding author(s): Wolfram Brune

Last updated by author(s): Dec 13, 2023

Reporting Summary

Nature Portfolio wishes to improve the reproducibility of the work that we publish. This form provides structure for consistency and transparency in reporting. For further information on Nature Portfolio policies, see our Editorial Policies and the Editorial Policy Checklist.

Statistics

For all statistical analyses, confirm that the following items are present in the figure legend, table legend, main text, or Methods section.

n/a	Confirmed	
☐	☒	The exact sample size (*n*) for each experimental group/condition, given as a discrete number and unit of measurement
☐	☒	A statement on whether measurements were taken from distinct samples or whether the same sample was measured repeatedly
☐	☒	The statistical test(s) used AND whether they are one- or two-sided<br>*Only common tests should be described solely by name; describe more complex techniques in the Methods section.*
☒	☐	A description of all covariates tested
☒	☐	A description of any assumptions or corrections, such as tests of normality and adjustment for multiple comparisons
☐	☒	A full description of the statistical parameters including central tendency (e.g. means) or other basic estimates (e.g. regression coefficient) AND variation (e.g. standard deviation) or associated estimates of uncertainty (e.g. confidence intervals)
☐	☒	For null hypothesis testing, the test statistic (e.g. *F*, *t*, *r*) with confidence intervals, effect sizes, degrees of freedom and *P* value noted<br>*Give P values as exact values whenever suitable.*
☒	☐	For Bayesian analysis, information on the choice of priors and Markov chain Monte Carlo settings
☒	☐	For hierarchical and complex designs, identification of the appropriate level for tests and full reporting of outcomes
☒	☐	Estimates of effect sizes (e.g. Cohen's *d*, Pearson's *r*), indicating how they were calculated

Our web collection on statistics for biologists contains articles on many of the points above.

Software and code

Policy information about availability of computer code

Data collection	Nikon NIS Elements 4.0 (Nikon)<br>Fusion Capture Advance FX7 16.15 (Peqlab)<br>FLUOstar Omega V1.30 (BMG Labtech)<br>HCS Studio Cell Analysis Software 4.0 (Thermo Fisher)<br>BD FACS Diva (BD Biosciences)
Data analysis	Nikon NIS Elements 4.51<br>NIH-ImageJ 1. 52i<br>GraphPad Prism 5.03<br>MS Excel<br>E-CRISP design tool, http://www.e-crisp.org/E-CRISP<br>CLC Genomics Workbench 7.9.1<br>FlowJo (version 10.8.1; Treestar)

For manuscripts utilizing custom algorithms or software that are central to the research but not yet described in published literature, software must be made available to editors and reviewers. We strongly encourage code deposition in a community repository (e.g. GitHub). See the Nature Portfolio guidelines for submitting code & software for further information.

Data

Policy information about availability of data

All manuscripts must include a data availability statement. This statement should provide the following information, where applicable:

- Accession codes, unique identifiers, or web links for publicly available datasets
- A description of any restrictions on data availability
- For clinical datasets or third party data, please ensure that the statement adheres to our policy

All data generated and analyzed during this study are included in the published article and its supplementary information files. Source data are provided with this paper.

Research involving human participants, their data, or biological material

Policy information about studies with human participants or human data. See also policy information about sex, gender (identity/presentation), and sexual orientation and race, ethnicity and racism.

Reporting on sex and gender	n/a
Reporting on race, ethnicity, or other socially relevant groupings	n/a
Population characteristics	n/a
Recruitment	n/a
Ethics oversight	n/a

Note that full information on the approval of the study protocol must also be provided in the manuscript.

Field-specific reporting

Please select the one below that is the best fit for your research. If you are not sure, read the appropriate sections before making your selection.

☒ Life sciences ☐ Behavioural & social sciences ☐ Ecological, evolutionary & environmental sciences

For a reference copy of the document with all sections, see nature.com/documents/nr-reporting-summary-flat.pdf

Life sciences study design

All studies must disclose on these points even when the disclosure is negative.

Sample size	Based on past experience, a minimum of 5 animals per group are required to detect statistically significant differences in MCMV infection experiments. For analysis of early MCMV replication in vivo (day 3 p.i.), five or six wild type mice were infected whith each virus strain (n=5 or 6). Seven ASC-/- mice were infected with each virus strain (n=7). For analysis of MCMV dissemination (day 14 p.i.), five wild type mice were infected with each virus strain (n=5).<br>Cell culture and in vitro experiments were performed with at least 3 biological replicates for statistical significance.
Data exclusions	No data points were excluded.
Replication	Results were reproduced by at least 3 independent experiments and all repeats were successful.
Randomization	For mouse experiments, females of the same genotype were randomly grouped into cages to exclude age variability (six to eight weeks-old). Randomization was not relevant for cell culture experiments as homogeneous cell populations (cell lines) were used.
Blinding	Scientists were not blinded to group/sample allocation in this study (not applicable, data are quantitative and do not require researcher judgement calls).

Reporting for specific materials, systems and methods

We require information from authors about some types of materials, experimental systems and methods used in many studies. Here, indicate whether each material, system or method listed is relevant to your study. If you are not sure if a list item applies to your research, read the appropriate section before selecting a response.

Materials & experimental systems

n/a	Involved in the study
☐	☒ Antibodies
☐	☒ Eukaryotic cell lines
☒	☐ Palaeontology and archaeology
☐	☒ Animals and other organisms
☒	☐ Clinical data
☒	☐ Dual use research of concern
☒	☐ Plants

Methods

n/a	Involved in the study
☒	☐ ChIP-seq
☐	☒ Flow cytometry
☒	☐ MRI-based neuroimaging

Antibodies

Antibodies used

Antibodies recognizing the following epitopes and proteins were used:
HA (Clone 3F10, Roche, #11867423001),WB dilution 1:1000, IF dilution 1:300
Flag (Clone M2, Sigma, #F3165), WB dilution 1:2500, IP dilution 1:300
IE1 (CROMA101, WB dilution 1:1000) were obtained from the Center for Proteomics, University of Rijeka
β-Actin (AC-15; Sigma, #A1978), WB dilution 1:10000
AIM2 (Cell Signaling; #63660), WB dilution 1:1000
ASC (D2W8U; Cell Signaling, #67824), WB dilution 1:1000
Caspase-1 (Casper-1; AdipoGen, AG-20B-0042-C100), WB dilution1:1000
GSDMD (EPR19828; Abcam, ab209845), WB dilution 1:1000
GFP (clones 7.1 and 13.1, Roche, #11814460001), WB dilution 1:1000
Secondary Antibodies:
Anti-Rat Alexa Fluor 488 (A-21208, Invitrogen) dilution 1:1000.
Anti-Mouse HRP (P044701-2, Dako), dilution 1:5000.
Anti-Rabbit HRP (P039901-2, Dako), dilution 1:5000.
Anti-Rabbit IgG heavy chain (ab99702, Abcam), dilution 1:4000.
Anti-Rat HRP (112-035-062, Jackson ImmunoResearch), dilution 1:5000.

Validation

All antibodies were obtained from commercial vendors. Validation of individual antibodies can be accessed via the following links:
HA (3F10), https://www.sigmaaldrich.com/US/en/product/roche/roahaha
Flag (M2), https://www.sigmaaldrich.com/US/en/product/sigma/f3165
IE1 (CROMA191), https://products.capri.com.hr/product/anti-m123-ie1-mcmv
β-Actin (AC-15), https://www.sigmaaldrich.com/US/en/product/sigma/a1978
AIM2 (#63660), https://www.cellsignal.com/products/primary-antibodies/aim2-antibody/63660
ASC (D2W8U), https://www.cellsignal.com/products/primary-antibodies/asc-tms1-d2w8u-rabbit-mab/67824
Caspase-1 (Casper-1), https://adipogen.com/ag-20b-0042-anti-caspase-1-p20-mouse-mab-casper-1.html
GSDMD (EPR19818), https://www.abcam.com/en-lb/products/primary-antibodies/gsdmd-antibody-epr19828-ab209845
GFP (7.1 and 13.1), https://www.sigmaaldrich.com/US/en/product/roche/11814460001
Anti-rat AlexaFluor488, https://www.thermofisher.com/antibody/product/Donkey-anti-Rat-IgG-H-L-Highly-Cross-Adsorbed-Secondary-Antibody-Polyclonal/A-21208
Anti-Mouse HRP (P044701-2), https://www.agilent.com/store/productDetail.jsp?catalogId=P044701-2
Anti-Rabbit HRP (P039901-2), https://www.agilent.com/store/productDetail.jsp?catalogId=P039901-2
Anti-Rabbit IgG heavy chain (ab99702), https://www.abcam.com/products/secondary-antibodies/mouse-monoclonal-2a9-rabbit-igg-heavy-chain-hrp-ab99702.html
Anti-Rat HRP (112-035-062), https://www.jacksonimmuno.com/catalog/products/112-035-062

Eukaryotic cell lines

Policy information about cell lines and Sex and Gender in Research

Cell line source(s)

M2-10B4 (CRL-1972), HEK-293T cells (CL-11268) and Phoenix-Ampho (CRL-3213) were obtained from the American Type Culture Collection.
HEK-293A cells (R705-07) were purchased from Invitrogen.
Murine J774A.1 macrophages (ECACC 91051511) were obtained from the European Collection of Authenticated Cell Cultures.
Immortalized bone marrow-derived macrophages (iBMDMs, NR-9456) were obtained from BEI Resources.
BMDMs expressing ASC-mCherry were isolated from transgenic male mice that were provided by Mathias Gelderblom (University Medical Center Hamburg-Eppendorf).
Murine 10.1 fibroblasts have been descibed (Harvey and Levine, 1991).
AIM2- and ASC-deficient iBMDMs were generated by using CRISPR/Cas9 gene editing technology.
iBMDMs stably expressing iGLuc were generated by retroviral transduction with pMSCV-puro-iGLuc.

Authentication

Cell lines were authenticated based on morphology.
Gene knockout lines were authenticated by the lack of the respective gene product (protein) by immunoblot.

Mycoplasma contamination	All cell lines were tested regularly by PCR for mycoplasma contamination. No contamination was detected.
Commonly misidentified lines (See ICLAC register)	No commonly misidentified cell lines were used in the study.

Animals and other research organisms

Policy information about studies involving animals; ARRIVE guidelines recommended for reporting animal research, and Sex and Gender in Research

Laboratory animals	Animals used in this study were 6 to 8 weeks old female mice (Mus musculus). Asc+/+ (wildtype, littermate controls) and Asc-/- (KO, Pycard tm1Vmd) mice were on a C57BL/6N background. When no comparison with KO mice was performed , C57BL/6J mice were used.
Wild animals	No wild animals were used in this study.
Reporting on sex	Only female mice were used, as stated in the Methods section. This is standard practice in the field.
Field-collected samples	No field collected samples were used in this study.
Ethics oversight	All animal experiments were performed according to the recommendations and guidelines of the FELASA (Federation for Laboratory Animal Science Associations) and Society of Laboratory Animals (GV-SOLAS) and approved by the institutional review board and local authorities (Behörde für Gesundheit und Verbraucherschutz, Amt für Verbraucherschutz, Freie und Hansestadt Hamburg, reference number N017/2019).

Note that full information on the approval of the study protocol must also be provided in the manuscript.

Flow Cytometry

Plots

Confirm that:

- [x] The axis labels state the marker and fluorochrome used (e.g. CD4-FITC).
- [x] The axis scales are clearly visible. Include numbers along axes only for bottom left plot of group (a 'group' is an analysis of identical markers).
- [x] All plots are contour plots with outliers or pseudocolor plots.
- [x] A numerical value for number of cells or percentage (with statistics) is provided.

Methodology

Sample preparation	HEK 293A cells in 6-well plates were transfected with plasmids encoding murine AIM2 (500 ng) and ASC-GFP or GFP (500 ng) together with 2.5 µg M83 or M84 or empty vector plasmid. 24 h post-transfection, cells were detached by trypsinization, washed in PBS, and fixed in 4% (w/v) paraformaldehyde at RT for 15 min. Fixed cells were pelleted and resuspended in 700 µL PBS. Samples were stored at 4 °C 0.5h-2h before the acquisition.<br>The number of ASC specks+ cells was quantified for at least 10000 GFP+ singlets cells.
Instrument	FACSCanto II (Beckton Dickinson)
Software	Data Collection: BD FACS Diva (BD Biosciences)<br>Data Analysis: FlowJo (version 10.8.1; Treestar)
Cell population abundance	n/a
Gating strategy	Cell debris were excluded using FSC-A and SSC-A. Doublets were excluded using FSC-A and FSC-H. Only the high GFP+ cells (GFP-A) were analyzed for the formation of specks (high GFP-A, low GFP-W).

- [x] Tick this box to confirm that a figure exemplifying the gating strategy is provided in the Supplementary Information.
